# Anticholinergic drug use in elderly people: A population- based study in Iran

**DOI:** 10.22088/cjim.12.4.593

**Published:** 2021

**Authors:** Kimia Raei, Atena Rahimi, Seyed Reza Hosseini, Ali Akbar Moghadamnia, Ali Bijani

**Affiliations:** 1 Faculty of Medicine, Babol University of Medical Sciences, Babol, Iran; 2 Cellular and Molecular Biology Research Center, Health Research Institute, Babol University of Medical Sciences, Babol, Iran; 3 Department of Pharmacology and Toxicology, Babol University of Medical Sciences, Babol, Iran; 4 Social Determinants of Health Research Center, Health Research Institute, Babol University of Medical Sciences, Babol, Iran

**Keywords:** Anticholinergic drug scale, Drug burden index, Elderly, Pharmacoepidemiology

## Abstract

**Background:**

Elderly people are in need of several drugs due to physiological changes and multiple chronic diseases. Studies have shown that anticholinergic drugs can cause cognitive impairment, reduced physical activity, and increased mortality in elderly population. Paying attention to the anticholinergic medication use in older adults can prevent the occurrence of adverse events and increase the quality of health care. This study was conducted to quantify exposure to anticholinergic medicines in older people in Amirkola.

**Methods:**

This study is a part of the comprehensive cohort project that was being conducted from 2011 on the case patients of 60 years and above that referred to the Amirkola Health Center. A total of 1532 individuals were included, of whom 54.9% were men. The drug information was obtained by observing the patient’s prescription and self-report questionnaires and collected data were analyzed by SPSS software. Exposure to anticholinergic medications was measured using the drug burden index-anticholinergic (DBI-Ach) and the anticholinergic drug scale (ADS).

**Results:**

Among the 1532 elderly people with an average age of 69.21 years, 29% had DBI>0 and 36.3% had ADS>0. Also, there was a significant correlation between DBI and ADS (R=0.758). In addition, there is a significant relationship between sex variable with DBI and ADS (P=0.0001). So, women in comparison with men had higher values of DBI and ADS.

**Conclusion:**

The findings of this study indicate that anticholinergic exposure is relatively high especially in older women, which posed special precautions to avoid inappropriate prescribing in the elderly.

Population ageing is increasing in many countries of the world. According to the 2016 census, more than 9.1 percent of Iran's population consisted of people aged 60 and over. Despite the fact that Iran's population is still young, it is expected to rapidly move to aging thus the number of its elders will be more than 26 million in 2050 and its proportion to the total population will reach about 23 percent (1-3). Elderly people are more likely to have multiple, concurrent chronic conditions which is why polypharmacy in the elderly is more than the younger adults that can result in negative physical or mental health outcomes (4-8). In the studies conducted in Iran in 2011, it was also shown that more than 70% of the elderly consumed at least one drug (9, 10). Anticholinergic drugs are widely prescribed in the older population. By virtue of their antagonistic activity on muscarinic receptors, anticholinergic agents block the neurotransmitter acetylcholine (Ach) in the peripheral and central nervous system (CNS). These classes of medicines are used to treat various medical conditions common in older people, e.g., antihistamines in allergies, antipsychotics in psychosis, behavioral disorders and dementia, antidepressants in depression, and ipratropium and tiotropium for respiratory diseases (11-13).

The use of medicines with anticholinergic activity in older people is a concern due to multiple complications of these drugs such as impaired physical function, loss of balance, cognitive impairment, delirium, dizziness, hallucination, dry mouth, blurred vision, constipation, urinary retention, delirium, and tachycardia (14-19). Concurrent use of several medications with anticholinergic properties, certain age-related changes in pharmacokinetics, permeability alteration in the blood-brain barrier (BBB), and changes in cholinergic neurons or receptor function make the aged population more vulnerable to adverse drug reactions (19-21). 

There are several screening tools that have been developed to estimate anticholinergic burden in the elderly population. Hilmer et al. published the most appropriate tool, DBI, in 2007 (22) to determine the overall exposure to anticholinergic and sedative drugs and assess the possible impact of this index on physical activity and cognitive function in older adults. The DBI implements the principles of pharmacologic dose–response effect and the daily dose of anticholinergic medications is considered in the model (23, 24). The daily dose of an anticholinergic and/or sedative medicine is taken by the individual and their minimum effective doses are used in the calculation of DBI. More recent evidence has demonstrated an association between higher DBI and a range of adverse effects such as reduced physical function, falls, infirmity, difficulties in activities of daily living (ADL) and other adverse health outcomes (25-28).

The ADS, a pharmacological risk assessment tool was published by Carnahan et al. to estimate anticholinergic drug burden. In this scale, drugs are classified into four levels based on pharmacological properties and anticholinergic activity: drugs are known to have no anticholinergic effects (ADS=0); drug with potential anticholinergic properties (ADS=1); drugs with anticholinergic side effects at high doses (ADS=2); and drugs with significant anticholinergic properties (ADS=3) (29). ADS total score was calculated by the sum of the ADS score for each individual drugs. For example, a total ADS score for a patient is taking aspirin (level 0), imipramine (level 3), furosemide (1), is 4. More recent evidence reveals that higher ADS scores are associated with adverse effects of anticholinergic medicines (12, 30-32). Since the elderly population in Iran, similar to global trends, is increasing, more aged people may be exposed to medications with anticholinergic effects. Recent evidence has revealed a correlation between close relationship between a higher DBI-Ach and ADS scores and exacerbation of physical and cognitive impairment in older adults. Therefore, the study of the use of anticholinergic drugs in the elderly population can help prevent these complications and improve the health status of this population. Our knowledge of the anticholinergic drugs burden in Iran is largely based on very limited data. The aim of this study was therefore to estimate the total exposure to anticholinergic medications in people aged above 60 years, enrolled in the Amirkola Health and Ageing Project (AHAP).

## Methods

**Study population:** This research came from the first phase of original cohort study of AHAP, that was conducted in Amirkola, northern Iran, from 2011 to 2012 and has been described in detail by Hosseini et al. (33). All individuals above 60 years of age who received at least one medicine were included in the study (n=1616). Data were collected based on a questionnaire containing demographic information (sex, age, education, and work status), medical conditions and medication exposure (name and number of medications used, duration of use) information for all AHAP participants through self-report as well as observing the prescription and non-prescription medicines. A total of 84 participants were excluded from analysis due to incomplete or missing data in one or more variables (daily dose, medication strength, number of diseases, age, and unknown gender).

**Calculating anticholinergic exposure:** In this study, anticholinergic exposure was calculated using the DBI-Ach and the ADS. The DBI for each drug with anticholinergic properties was computed in accordance with the following formula, where D is the daily dose of an anticholinergic medication that was taken by the individual and δ is the minimum effective daily dose (approved by the Food and Drug Administration) (22). 

DBI_ACh_= ∑ D/(D+δ)

Both prescription and over-the-counter (OTC) medicines were included in the analysis. The minimum effective daily dose for each drug was determined by the British National Formulary (BNF) 2017. Finally, individuals were classified into three to three different levels of drug burden index ranges: DBI=0 (no exposure), DBI<1 (low exposure), and DBI≥1 (high exposure) (26). Anticholinergic drug exposure was assessed using the ADS as well. So, medications with anticholinergic effects were extracted and rated using the ADS and an individual's ADS total score was calculated as the sum of the scores for all prescribed anticholinergic medications (34). Finally the participants were divided into five subgroups based on overall ADS scores: group with total ADS score <3, group with total ADS score =3, group with total ADS score=4, group with total ADS score =5, and group with total ADS score ≥6 (30). 

**Statistical Analysis:** Data were statistically analyzed using the Statistical Package for the Social Sciences (SPSS 23.0, SPSS Inc., USA) and statistical tests such as t-test, chi-square, Pearson correlation, and logistic regression. A p-value less than 0.05 was considered as a significant threshold.

## Results

The AHAP study consisted of 1616 individuals aged 60 years and older. The mean age (±SD) of participants was 69.21±7.35 years (range 60–92), 54.9% of whom were men and 45.1% were women (table 1). At baseline, the mean number of all regular medications per participant was 2.78±2.69 and the mean total DBI and ADS in the study sample of 1532 were 0.22±0.44 and 1.01±1.99, respectively (table 1). As shown in table 2, the DBI medications used most frequently by the participants were ranitidine (109 patients exposed), followed by alprazolam (75 patients exposed), clidinium-C (56 patients exposed), furosemide (42 patients exposed), and digoxin (29) patients exposed. Additional analysis revealed that amitriptyline (23 patients exposed), nortriptyline (17 patients exposed), and imipramine (11 patients exposed) were the most commonly anticholinergic medications for an ADS level of 3, whereas among the medicines with ADS level of 2 and 1, ranitidine (109 patients exposed) and alprazolam (75 patients exposed) were more frequently prescribed in participants, respectively (table 3). A significant difference between the scales was revealed. 

As shown in table 4, based on DBI scale, the majority of participants in the study had no anticholinergic drug exposure, while around 29% had DBI score>0. Furthermore, of the 1532 participants, 1319 (86.1%) had an ADS sum score <3, 213 (13.9%) had an ADS sum score ≥3 and among them, the number of people who had ADS sum score ≥6 was the most (n=79). In table 5, we showed that DBI score, ADS score, number of diseases and number of prescribed drugs are remarkably higher in females in comparison with males (p<0.001). No significant association appeared between ADS and DBI score with age and level of education that was not significant (data not shown).

**Table 1:** Characteristics of the study population (n =1532)

**Characteristics **	
Average age in years (±SD) Range (years)	69.21±7.35 60-92
Gender N (%) Female Male	691 (45.1) 841 (54.9)
Average number of prescribed medicines (±SD)	2.78±2.69
Exposed to at least one anticholinergic drug with an ADS score (%)	29
Exposed to at least one anticholinergic drug using DBI-Ach (%)	36.3

ADS, Anticholinergic Drug Scale; DBI-Ach, Drug Burden Index-Anticholinergic component

**Table 2:** Most frequently used DBI medicines in the study population (n = 1532)

**Drug **	**Frequency, n (%)**
Ranitidine	109 (16.4)
Alprazolam	75 (11.3)
Clidinium-C	56 (8.4)
Furosemide	42 (6.3)
Digoxin	29 (4.3)
Gabapentin	23 (3.4)
Amitriptyline	23 (3.4)
Diltiazem	22 (3.3)
Trifluoperazine	18 (2.7)
Nortriptyline	17 (2.5)

**Table 3:** Most frequently used ADS medicines in the study population (n = 1532)

** ADS = 1 ADS = 2 ADS = 3**				
Drug Frequency, n (%)	Drug	Frequency,n(%)	Drug	Frequency, n (%)
Alprazolam 75 (11.3)	Ranitidine	109 (16.4)	Amitriptyline	23 (3.4)
Triamterene 53 (7.8)	Cimetidine	8 (1.1)	Nortriptyline	17 (2.5)
Furosemide 42 (6.2)	Cyproheptadine	4 (0.5)	Imipramine	11 (1.6)
Digoxin 29 (4.2)	Carbamazepine	2 (0.2)	Dimenhydrinate	8 (1.1)
Lorazepam 27 (4)			Trihexyphenidyl	7 (1)

ADS, Anticholinergic Drug Scale

**Table 4:** Distribution of participants (n=1532) based on degree of anticholinergic drug exposure for each scale

**Scale **	**Frequency, n (%)**
DBI	
0	1088 (71)
<1	327 (21.4)
≥1	117 (7.6)
ADS	
<3	1319 (86.1)
3	49 (3.2)
4	59 (3.8)
5	26 (1.7)
≥6	79 (5.2)

ADS, Anticholinergic Drug Scale; DBI, Drug Burden Index

**Table 5:** Relationship between variables with gender in the study population (n = 1532)

	**Gender**	**Mean ± SD**	**P value**
DBI	Male Female	0.15±0.38 0.31±0.48	<0.001
ADS	Male Female	0.68±1.64 1.42±2.29	<0.001
Number of diseases	Male Female	2.13±1.74 3.39±1.98	<0.001
Number of prescribed medicines	Male Female	2.1±2.45 3.39±1.98	<0.001
Age (years)	Male Female	69.83±7.62 68.47±6.94	<0.001

ADS, Anticholinergic Drug Scale; DBI, Drug Burden Index

## Discussion

The finding of this study indicates that a significant percentage of participants used at least one medicine with anticholinergic effects during the study. The prevalence of anticholinergic exposure was estimated at around 29% using the DBI-ACh scale and 36.3 % with ADS. Interestingly, gender differences exist in exposure to anticholinergic medicines in a population study, hence, women were more likely to be exposed to drugs with anticholinergic medicines than men. The number of medications included in each scale differs and it can justify why the ADS identified a larger proportion of older adults exposed to agents with anticholinergic properties in comparison with the DBI (35). Various studies have been reported with regard anticholinergic exposure in older adults. The results of these studies varied based on study design, type of assessment tool, population health status and their administered medications (36). 1n 2013 Narayan et al. in New Zealand showed that among 537,387 people aged 65 years and above were included in the study, 31.80 % and 52.66 % of older adults were exposed to anticholinergic medications as determined by the DBI and the ADS, respectively (12). The study by Nishtala et al. (11) found that amongst the study population, 43.22% were exposed to at least one anticholinergic medicine based on the DBI scale. 

In 2017, Lampela et al. assessed the anticholinergic exposure in population aged 75 years and older using the four ranked anticholinergic lists. They reported that about 57% of individuals use at least one anticholinergic drug according to the ADS. The mean anticholinergic score was higher when using ACB (anticholinergic cognitive burden scale), while the anticholinergic exposure was lower as defined by ARS (anticholinergic risk scale) or Chew's list (37). The evaluation of anticholinergic burden in patients with advanced chronic conditions who are admitted to an acute care hospital showed that 93.6% and 82.10% of the patients were exposed with anticholinergic medications as determined by the ADS and the DBI, respectively. Our results do not appear to corroborate these findings due to difference in population study (general population compared with individuals with chronic diseases) and average number of drugs (2.78 compared with 9.46) (38). 

The results of this study have further strengthened our hypothesis that women are highly exposed to medications with anticholinergic properties than men. Although in our study, the men population was greater than that of women, exposure to anticholinergic medications based on both DBI-Ach and ADS was significantly higher in women that could be interpreted as being a result of multiplicity of drugs in female population and this fact means the number of chronic diseases in older women was more than in men in Amirkola (39). This concurs well with previous findings (11, 38).

Our research may have some limitations. Some participants may have used over-the-counter anticholinergic medications during the study that could not be included in the dataset. Moreover, in spite of the special attention that has been made to identify the actual drug use, over- and under-reporting of medication utilization may have occurred by some individuals. Another limitation is that, participants’ adherence to medication regimen was not clear for researchers. In addition, it could not be precisely determined whether the dispensed medications were consumed and for how long they used the medicines.

In conclusion, on a population level, the evidence from this study has intimated the extensive use of medicines with anticholinergic effects in this population of older adults. In addition, women were more probable to be exposed to anticholinergic medicines than men. Considering the broad use of anticholinergic medicines in older population and their adverse effects, further studies are needed to determine the association between anticholinergic drug use and related consequences in the older adults.

## References

[1] Hosseini S, Keshavarz A, Amin A (2010). Nutritional status and non-diet associated factors of hospitalized heart-failure elderly patients. Iran J Ageing.

[2] Russel M, Ardalan A (2007). The future of ageing and its health care costs: a warning for health system. Iran J Ageing.

[3] Bagheri-Nesami M, Hamzehgardeshi Z (2013). Experiencing the onset of aging: a qualitative study. J Mazandaran Univ Med Sci.

[4] Barry PJ, Gallagher P, Ryan C (2008). Inappropriate prescribing in geriatric patients. Curr Psychiatry Rep.

[5] Fulton MM, Riley Allen E (2005). Polypharmacy in the elderly: a literature review. J Am Acad Nurse Pract.

[6] Cherubini A, Ruggiero C, Gasperini B (2011). The prevention of adverse drug reactions in older subjects. Curr Drug Metab.

[7] Tamura BK, Bell CL, Inaba M, Masaki KH (2012). Outcomes of polypharmacy in nursing home residents. Clin Geriatr Med.

[8] Zed PJ, Abu-Laban RB, Balen RM (2008). Incidence, severity and preventability of medication-related visits to the emergency department: a prospective study. CMAJ.

[9] Delshad Noghabi A, Baloochi Beydokhti T (2013). Polypharmacy and its related factors among elderlies. Iran J Nursing.

[10] Heidari S, Gholizadeh LM, Asadolahi F, Abedini Z (2013). Evaluation of health status of elderly in Qom city, 2011, Iran. Qom Univ Med Sci J.

[11] Nishtala PS, Narayan SW, Wang T, Hilmer SN (2014). Associations of drug burden index with falls, general practitioner visits, and mortality in older people. Pharmacoepidemiol Drug Saf.

[12] Narayan SW, Hilmer SN, Horsburgh S, Nishtala PS (2013). Anticholinergic component of the Drug Burden Index and the Anticholinergic Drug Scale as measures of anticholinergic exposure in older people in New Zealand: a population-level study. Drugs Aging.

[13] Green AR, Reifler LM, Bayliss EA, Weffald LA, Boyd CM (2019). Drugs contributing to anticholinergic burden and risk of fall or fall-related injury among older adults with mild cognitive impairment, dementia and multiple chronic conditions: a retrospective cohort study. Drugs Aging.

[14] Lowry E, Woodman RJ, Soiza RL, Hilmer SN, Mangoni AA (2012). Drug burden index, physical function, and adverse outcomes in older hospitalized patients. J Clin Pharmacol.

[15] Kumpula EK, Bell JS, Soini H, Pitkälä KH (2011). Anticholinergic drug use and mortality among residents of long‐term care facilities: a prospective cohort study. J Clin Pharmacol.

[16] Ambrose AF, Paul G, Hausdorff JM (2013). Risk factors for falls among older adults: a review of the literature. Maturitas.

[17] Bennett A, Gnjidic D, Gillett M, Carroll P, Matthews S, Johnell K (2014). Prevalence and impact of fall-risk-increasing drugs, polypharmacy, and drug–drug interactions in robust versus frail hospitalised falls patients: a prospective cohort study. Drugs Aging.

[18] Farrell B, Eisener-Parsche P, Dalton D (2014). Turning over the rocks: Role of anticholinergics and benzodiazepines in cognitive decline and falls. Can Fam Physician.

[19] Pasina L, Colzani L, Cortesi L (2019). Relation between delirium and anticholinergic drug burden in a cohort of hospitalized older patients: an observational study. Drugs Aging.

[20] Bostock CV, Soiza RL, Mangoni AA (2010). Association between prescribing of antimuscarinic drugs and antimuscarinic adverse effects in older people. Expert Rev Clin Pharmacol.

[21] Mangoni AA, Jackson SH (2004). Age‐related changes in pharmacokinetics and pharmacodynamics: basic principles and practical applications. Br J Clin Pharmacol.

[22] Hilmer SN, Mager DE, Simonsick EM (2007). A drug burden index to define the functional burden of medications in older people. Arch Intern Med.

[23] Taipale HT, Hartikainen S, Bell JS (2010). A comparison of four methods to quantify the cumulative effect of taking multiple drugs with sedative properties. Am J Geriatr Pharmacother.

[24] Cardwell K, Hughes CM, Ryan C (2015). The association between anticholinergic medication burden and health related outcomes in the ‘oldest old’: a systematic review of the literature. Drugs Aging.

[25] Gnjidic D, Bell JS, Hilmer SN (2012). Drug burden index associated with function in community-dwelling older people in finland: a cross-sectional study. Ann Med.

[26] Gnjidic D, Cumming RG, Le Couteur DG (2009). Drug Burden Index and physical function in older Australian men. Br J Clin Pharmacol.

[27] Hilmer SN, Mager DE, Simonsick EM (2009). Drug burden index score and functional decline in older people. Am J Med.

[28] Wouters H, Hilmer SN, Twisk J (2020). Drug Burden Index and Cognitive and Physical Function in Aged Care Residents: A Longitudinal Study. J Am Med Dir Assoc.

[29] Carnahan RM, Lund BC, Perry PJ, Pollock BG, Culp KR (2006). The Anticholinergic Drug Scale as a measure of drug‐related anticholinergic burden: Associations with serum anticholinergic activity. J Clin Pharmacol.

[30] Kersten H, Molden E, Willumsen T, Engedal K, Wyller TB (2013). Higher anticholinergic drug scale (ADS) scores are associated with peripheral but not cognitive markers of cholinergic blockade Cross sectional data from 21 Norwegian nursing homes. Br J Clin Pharmacol.

[31] Ruxton K, Woodman RJ, Mangoni AA (2015). Drugs with anticholinergic effects and cognitive impairment, falls and all‐cause mortality in older adults: a systematic review and meta‐analysis. Br J Clin Pharmacol.

[32] Lee MS, Hanger HC (2017). Audit of anticholinergic medication changes in older hospitalised patients using the anticholinergic drug scale. Intern Med J.

[33] Hosseini SR, Cumming RG, Kheirkhah F (2013). Cohort profile: The Amirkola health and ageing project (AHAP). Int J Epidemiol.

[34] Carnahan R, Lund B, Perry P, Culp KR, Pollock B (2002). The relationship of an anticholinergic rating scale with serum anticholinergic activity in elderly nursing home residents. Psychopharmacol Bull.

[35] Naples JG, Marcum ZA, Perera S (2015). Concordance between anticholinergic burden scales. J Am Geriatr Soc.

[36] Campbell N, Perkins A, Hui S, Khan B, Boustani M (2011). Association between prescribing of anticholinergic medications and incident delirium: a cohort study. J American Geriatr Soc.

[37] Lampela P, Taipale H, Lavikainen P, Hartikainen S (2017). The effect of comprehensive geriatric assessment on anticholinergic exposure assessed by four ranked anticholinergic lists. Arch Gerontol Geriatr.

[38] Sevilla‐Sánchez D, Molist‐Brunet N, González‐Bueno J (2018). Prevalence, risk factors and adverse outcomes of anticholinergic burden in patients with advanced chronic conditions at hospital admission. Geriatr Gerontol Int.

[39] Hosseini SR, Cumming RG, Sajjadi P, Bijani A (2011). Chronic diseases among older people in Amirkola, northern Islamic Republic of Iran. East Mediterr Health J.

